# Teachers’ Affective Well-being and Teaching Experience: The Protective Role of Perceived Emotional Intelligence

**DOI:** 10.3389/fpsyg.2017.02227

**Published:** 2017-12-19

**Authors:** Pablo Fernández-Berrocal, María J. Gutiérrez-Cobo, Juan Rodriguez-Corrales, Rosario Cabello

**Affiliations:** ^1^Department of Basic Psychology, Faculty of Psychology, University of Málaga, Málaga, Spain; ^2^Department of Developmental and Educational Psychology, University of Granada, Granada, Spain

**Keywords:** teachers, affective well-being, positive affect, perceived emotional intelligence, teaching experience

## Abstract

Teaching is a highly emotional and demanding profession. Developing emotional well-being among teachers will benefit not only the teachers themselves, but also their students. Previous studies have shown the protective role of emotional intelligence (EI) as well as inconsistencies in the years of teaching experience variable on positive and negative work-specific variables. The aim of the present study was to analyze how EI and years of teaching experience are related to affective well-being in teachers. Further, we analyze the moderator role of perceived EI on the link between level of teaching experience and affective well-being. For these purpose, 524 teachers from different Spanish public schools took part in the study. They first completed the Trait Meta-Mood Scale-24 (TMMS-24) for measuring perceived EI, which evaluates three scales: Attention to one’s Feelings (Attention), Emotional Clarity (Clarity), and Mood Repair (Repair). Secondly, they completed the Positive and Negative Affect Schedule (PANAS) for affective well-being, which measures Positive Affect (PA) and Negative Affect (NA). Finally, teachers indicated their years of teaching experience. The results revealed that teaching experience and attention variables are counterproductive in determining lower PA and higher NA, respectively. Clarity and Repair appeared to be a significant determinant of PA and NA, with higher Clarity and Repair determining higher PA and lower NA. Moderator analyses showed how teaching experience significantly decreased PA in teachers who had average or low levels of Repair, but not for those with higher levels of this variable, emphasizing the important role of Repair as a protector of affective well-being in teachers. Limitations and future areas for research are discussed.

## Introduction

The workplace can be the focus of stress and mental health problems as a consequence of issues that include high emotional or cognitive demands, lack of opportunities, workload, or feeling undervalued (Eurofond, 2012; American Psychological Association, 2013; Beehr, 2014). Improving well-being among employees has benefits not only for the employees themselves, but also for the success of the organization (Giorgi et al., 2017). Therefore, given this shared benefit, there has been an increasing interest in looking for protective factors and interventions that promote well-being in the occupational setting (Biron and Karanika-Murray, 2013; Shropshire and Kadlec, 2015).

Among the multiple organizations under study, the literature has been paying increasing attention to the well-being of teachers. Teaching is a highly emotional profession associated with high levels of stress that may be the cause of job dissatisfaction, psychological disorders, and reduced well-being (Chang, 2009; Brackett et al., 2010; Keller et al., 2014). In addition, given the pivotal role of teachers in student issues and life learning, the emotional well-being of teachers is essential not only for themselves, but for their students (Frenzel et al., 2009; Jiang et al., 2016; Lee et al., 2016).

Subjective well-being is defined as “a person’s cognitive and affective evaluations of his or her life” (Diener et al., 2003; p. 63) and is composed of an affective and cognitive component. Affective well-being is defined as the prevalence of positive affect (PA) over negative affect (NA) (Kahneman et al., 1999); while cognitive well-being refers to the individuals’ evaluation of their past and present life (Pavot and Diener, 2008). Both components of subjective well-being are related to positive outcomes such as better health and longevity (Diener and Chan, 2011) and adequate regulation strategies (Szczygieł and Mikolajczak, 2017), with affective well-being considered by some researchers as more relevant for well-being (Kong and Zhao, 2013; Szczygieł and Mikolajczak, 2017). In addition, well-being is also vital for the teachers daily performance at work (Frenzel et al., 2016). Thus, certain interventions based on the introduction of proactive changes in the workplace for different types of employees — including teachers — have had the effect of enhancing their well-being and performance at work (Bakker et al., 2016; Hodzic et al., 2017; van Wingerden et al., 2017). Thus, it would be essential to look for mechanisms for improving affective well-being among these workers.

Emotional intelligence (EI) is an important concept that has been the focus of numerous investigations, given its link with positive work-related outcomes (Côté, 2014; Lopes, 2016). EI is defined as the integration of the ability to perceive, appraise, and express emotion accurately; the ability to access, and/or generate feelings when they facilitate thought; the ability to understand emotion and emotional knowledge; and the ability to regulate emotions to promote emotional and intellectual growth (Mayer and Salovey, 1997; Mayer et al., 2016). EI can improve well-being in the general population (Cabello and Fernández-Berrocal, 2015b; Fernández-Berrocal and Extremera, 2016) as well as in the employees within the organizational setting (Di Fabio and Kenny, 2016). With regard to teachers, the majority of the literature has focused on analyzing the role of EI in reducing negative work-specific variables such as burnout, or increasing positive variables such as job satisfaction and engagement (Yin et al., 2013; Sun et al., 2017; for a review see Mérida-López and Extremera, 2017). However, although these work-specific variables are related and can influence well-being, they are distinct constructs. In addition, some studies have gone a step further by showing how EI not only benefits specific work-related variables, but also more general variables such as the cognitive well-being of teachers (Brackett et al., 2010; Vesely et al., 2014; Lee et al., 2016). However, rather less is known about the relationship between EI and affective well-being in schoolteachers. One exception is a study by Landa et al., 2006 with university teachers, which demonstrated how perceived EI was related to affective well-being in this population.

Traditionally, approaches to the study of EI fall into two main categories according to the type of instrument employed for its measurement. On the one hand, ability EI makes use of performance tests where individuals solve emotional problems in an objective manner with correct and incorrect responses (Mayer et al., 2002) and, on the other hand, perceived EI uses self-report tests where participants estimate their own EI (Salovey et al., 1995). In the present study, we will focus on perceived EI, given its strong association with affective well-being (Sánchez-Álvarez et al., 2015). Specifically, we will use the Trait Meta-Mood Scale (TMMS; Salovey et al., 1995), which provides information about perceived EI by evaluating three dimensions: Attention to one’s Feelings (Attention), Emotional Clarity (Clarity), and Mood Repair (Repair). Studies using TMMS have shown that Clarity and Repair are positively linked to PA and negatively correlated with NA (Palmer et al., 2002; Sánchez-Álvarez et al., 2015) acting as possible shock-absorbers of harmful situations. Although a moderate amount of attention may be useful in permitting people to check the progress of their moods and should be associated with PA, the persistent monitoring of feelings and moods may lead to a ruminative and self-focused process, which in turn implies higher immersion in NA (Lischetzke and Eid, 2003; Fernández-Berrocal and Extremera, 2008; Lischetzke et al., 2012; Kong and Zhao, 2013).

Another interesting source of the well-being of teachers is the number of years of teaching experience. An increased number of years of teaching appear to be beneficial for certain aspects of well-being, but detrimental for others. For instance, Kini and Podolsky (2016) found positive consequences of greater experience by associating it with teacher effectiveness. Nonetheless, previous investigations have found inconsistencies between the effects of the amount of years of teaching on well-being, with some finding positive correlations, and others negative or even no correlations (Taylor and Tashakkori, 1995; Xin and MacMillan, 1999; Kristiansen et al., 2011; Gastaldi et al., 2014; Kourmousi and Alexopoulos, 2016). Thus, there is a need to clarify the effects of the years of teaching variable on teachers’ affective well-being.

Given the importance of knowing the factors that influence the teacher affective well-being, the aims of the present study were two-fold: (1) To evaluate the relationship between years of teaching experience and perceived EI on the affective well-being of teachers, and (2) to analyze the moderator role of EI on the relationship between teaching experience and affective well-being.

More specifically, we propose the following hypotheses. First, we expected that, as indicated by the literature, years of teaching experience would be negatively associated with PA and positively associated with NA. Second, we expected that Attention would be positively related to NA, and both Clarity and Repair would be positively associated with PA, but negatively associated with NA. Third, we expected that Clarity and Repair would moderate the relationship between teaching experience and affective well-being such that, in line with previous research, the associations between teaching experience and PA and NA would be significantly weaker for teachers with comparatively higher rather than lower levels of Clarity and Repair.

## Materials and Methods

### Participants and Procedure

The participants were 524 teachers (72.3% women) from 42 Andalusian (Spain) public schools who attended a series of training courses on SEL sponsored by the Regional Government of Andalusia and the authors’ laboratory. The age of the teachers ranged between 21 and 62 years (*M* = 39.07; *SD* = 9.48). In our sample, 22.2% taught elementary (3 to 6 year-old students), 45.7% taught primary (6 to 12 year old students), and 32.1% taught secondary school (12 to 16 year-old students). Teachers reported an average of almost 11 years of teaching experience (*SD* = 10.08; ranged between 1 and 36 years). Teachers who attended this series of SEL courses completed the study questionnaires for 10 min in a quiet room, and before the course began to avoid the possibility of the course content biasing responses to the questionnaire.

The study was carried out in accordance with the Declaration of Helsinki and ethical guidelines of the American Psychological Association, and all participants provided written informed consent. The Research Ethics Committee of the University of Málaga approved the study protocol as part of the projects SEJ-07325.

### Measures

*Trait Meta-Mood Scale-24* (TMMS-24) (Fernandez-Berrocal et al., 2004; original version Salovey et al., 1995) was designed to assess how people reflect upon their moods and provides an indicator of the levels of perceived EI. Respondents are asked to rate their degree of agreement on each of the 24 items on a 5-point Likert-type scale ranging from 1 (very much agree) to 5 (very much disagree). The scale is composed of three sub-factors: Attention to one’s Feelings (Attention), Emotional Clarity (Clarity), and Mood Repair (Repair). Attention, assessed by the first 8 items, is the degree to which people believe they pay attention to their own feelings (i.e., “I think about my mood constantly”); Clarity, evaluated by the following eight items, refers to how people believe that they perceive their emotions (i.e., “I am usually very clear about my feelings”), and Repair, assessed by the remaining 8 items, refers to people’s belief in their capacity to block negative moods and prolong positive moods (i.e., “Although I sometimes feel sad, I usually have an optimistic outlook”). Fernandez-Berrocal et al. (2004) found a high internal consistency (Cronbach’s alpha for Attention = 0.90, Clarity = 0.90, Repair = 0.86) and satisfactory test-retest reliability (*r* values ranging from 0.60 to 0.83), improving the psychometric properties of the original extended 48-item version (Cronbach’s alpha for Attention = 0.86, Clarity = 0.87, Repair = 0.82; Salovey et al., 1995).

The Spanish translation of the Positive and Negative Affect Schedule was used (PANAS; Watson et al., 1988; Sandín et al., 1999). The 20-item inventory consists of 10 adjectives measuring Negative Affect (NA: hostile, irritable, jittery, nervous, scared, afraid, ashamed, distressed, guilty, and upset) and 10 adjectives measuring Positive Affect (PA: excited, inspired, interested, proud, active, alert, attentive, determined, enthusiastic, and strong). Items were administered in the general format with the instructions to rate “*to what extent you generally feel this way*” on a scale ranging from 1 (very slightly or not at all) to 5 (extremely). PANAS has shown adequate psychometric properties in both the original version (Cronbach’s alpha for PA = 0.87, NA = 0.88), and the Spanish version (alphas ranged from 0.87 to 0.91; Sandín et al., 1999).

### Data Analyses

**FIGURE 1:**
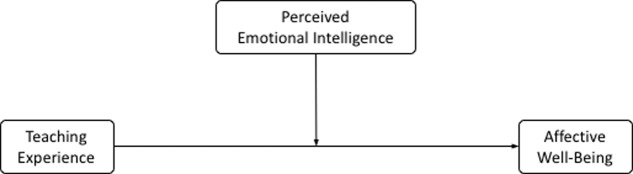
The conceptual moderation model.

All statistical analyses were carried out using the SPSS package (version 20.0; IBM, United States). Preliminary analyses were conducted to compute descriptive statistics. The analysis of the relationship between Gender, Teaching experience, Attention, Clarity, Repair, PA, and NA variables was conducted with Pearson’s coefficients and Kendall’s Tau-b. To investigate the strength of teaching experience and perceived EI as determinants of affective well-being, we conducted three-step hierarchical regression analyses. Gender was included as a control variable. We next used the moderation model from the PROCESS macro for SPSS (Hayes, 2013) to test the moderation hypothesis (**Figure 1**).

## Results

### Preliminary Analyses

Descriptive statistics and correlations among study variables are shown in **Table 1**. For all variables, skewness and kurtosis values were within an acceptable range of ±2 (Gravetter and Wallnau, 2014).

**Table 1:** Descriptive statistics and inter-correlations among the measures.

	*M*	SD	1	2	3	4	5	6	Alpha
1. Gender (0 = males)									
2. Teaching experience	11.31	10.10	-0.05						
3. Attention	3.31	0.79	0.06	–0.06					0.87
4. Clarity	3.47	0.71	0.01	–0.00	0.09^∗^				0.87
5. Repair	3.57	0.76	0.03	0.02	0.02	0.48^∗∗^			0.85
6. Positive Affect	3.62	0.67	–0.05	–0.19^∗∗^	–0.02	0.31^∗∗^	0.36^∗∗^		0.87
7. Negative Affect	2.15	0.72	0.08	0.03	0.30^∗∗^	–0.18^∗∗^	–0.22^∗∗^	–0.23^∗∗^	0.87

^∗^*p < 0.05; ^∗∗^*p* < 0.01*.

Teaching experience was negatively correlated with PA but not with NA. Otherwise, teaching experience showed no significant correlation with Attention, Clarity, or Repair. In addition, Attention was positively associated with NA, and higher Clarity and Repair were positively associated with PA and negatively associated with NA. Finally, there were positive correlations between Attention and Clarity, and between Clarity and Repair.

### Analyses of Hierarchical Regression

To determine the strength of teaching experience and perceived EI as determinants of affective well-being, we conducted two three-step hierarchical regression analyses. The determinant variables were gender, teaching experience, Attention, Clarity, and Repair while the criterion variables were PA and NA. We conducted the regressions by first entering gender into the model, followed by teaching experience, and finally Attention, Clarity, and Repair.

The results of the regression models are shown in **Table 2**. In the first analysis, gender was not a significant determinant of PA. Teaching experience, added at the second step of the models, proved to be a significant determinant of PA (Δ*R*^2^ = 0.04), with teaching experience predicting lower PA. In the third step of the model, Clarity and Repair were revealed to be significant determinants of PA (Δ*R*^2^ = 0.16) over and above the effects of teaching experience, with higher Clarity and Repair determining higher PA.

**Table 2:** Hierarchical regression to determine teachers’ affective well-being on the basis of teaching experience and perceived emotional intelligence (EI).

Determinant variables	Criterion variables
	
	Positive affect
	
	*B*	SE	β	*R*^2^	Δ*R*^2^	*F*(df)
Step 1				0.00	0.00	1.56 (1,522)
Gender	-0.08	0.06	-0.05			
Step 2				0.04***	0.04***	11.50 (2,521)^∗∗∗^
Gender	-0.10	0.06	-0.06			
Teaching experience	-0.01	0.00	-0.20***			
Step 3				0.20***	0.16***	26.61 (5,518)^∗∗∗^
Gender	-0.11	0.06	-0.12			
Teaching experience	-0.01	0.00	-0.21***			
Attention	-0.05	0.03	-0.06			
Clarity	0.17	0.04	0.18***			
Repair	0.25	0.04	0.28***			
Step 4				0.21***	0.01***	18.72 (8,515)^∗∗∗^
Gender	-0.13	0.06	-0.08			
Teaching experience	-0.01	0.00	-0.21***			
Attention	-0.05	0.03	-0.06			
Clarity	0.16	0.04	0.17***			
Repair	0.28	0.04	0.31***			
Teaching experience × Attention	-0.00	0.00	-0.07			
Teaching experience × Clarity	-0.00	0.00	-0.06			
Teaching experience × Repair	0.01	0.00	0.15***			
Total *R^2^*				0.21***		

**Negative affect**		

Step 1				0.00	0.00	3.22 (1,522)
Gender	0.13	0.07	0.08			
Step 2				0.00	0.00	1.95 (2,521)
Gender	0.13	0.07	0.08			
Teaching experience	0.00	0.00	0.04			
Step 3				0.16***	0.16***	20.19 (5,518)^∗∗∗^
Gender	0.11	0.06	0.07			
Teaching experience	0.00	0.00	0.06			
Attention	0.29	0.04	0.31***			
Clarity	-0.13	0.05	-0.12**			
Repair	-0.16	0.04	-0.17***			
Total *R^2^*				0.16***		

^∗∗^*p < 0.01; ^∗∗∗^*p* < 0.001*.

In the second analysis, Gender (Step 1) and Teaching experience (Step 2) were not significant determinants of NA. In the third step of the model, Attention and Clarity and Repair were shown to be significant determinants of NA (Δ*R*^2^ = 0.16), with higher Attention determining higher NA, and with higher Clarity and Repair determining lower NA.

### Moderation Analyses

**FIGURE 2:**
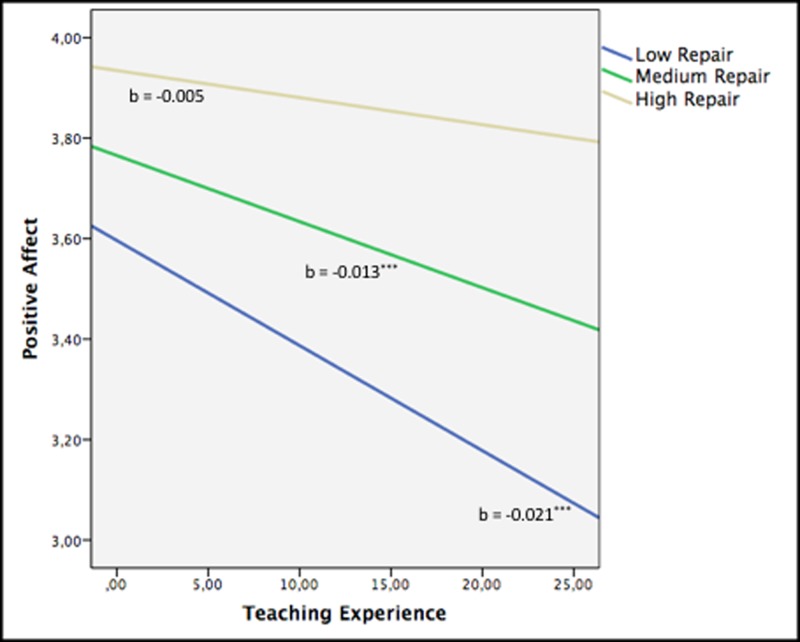
Moderation analysis with Repair as moderator on the association between teaching experience and positive affect. ^∗∗∗^*p* < 0.001.

We used Teaching experience, Attention, and Clarity and Repair as determinant variables and PA as the criterion factor (**Figure 2**). Gender was included as a control variable. We tested all two-way interactions between teaching experience and perceived EI (teaching experience *×* Attention, teaching experience *×* Clarity, teaching experience *×* Repair). A moderation analysis conducted on PA revealed a significant interaction between teaching experience and Repair (β = 0.15, *p* < 0.001; Δ*R*^2^ = 0.01), although the other two-way interactions between these variables were not significant (see Step 4 in **Table 2**).

**Figure 2** shows regions of significance for PA using the Johnson–Neyman technique. Teaching experience significantly decreased PA in teachers who had average or low levels of Repair. This variable began to exert a moderating effect at Repair below 4.20 (*t* = –1.96, *p* < 0.05). In particular, teachers with medium or low Repair scores below 4.20 obtained lower PA scores (3.48 and 3.20, respectively) than the mean score for teachers with high Repair (3.76).

These moderation analyses were not conducted for NA, since the previous analyzes did not show an association between teaching experience and NA.

## Discussion

In light of the many studies that have aimed to evaluate the relationship between EI and specific work-related variables, the aim of the current study was to analyze how the perceived EI and years of teaching experience variables were related to affective well-being in teachers. Further, and of more interest, we analyzed the moderator role of perceived EI in the relationship between teacher experience and affective well-being, to clarify its protective role.

Correlational and multiple regression analyses showed how the two variables of interest were related to different measures of affective well-being. Specifically, and consistent with our first hypothesis and the results of previous research (Taylor and Tashakkori, 1995; Gastaldi et al., 2014), longer teaching experience was a determinant of a reduction in PA among teachers. However, we failed to find any association with NA as predicted in the first hypothesis. On the other hand, and consistent with the second hypothesis, the different dimensions of perceived EI were also related to PA and NA. Thus, while Attention to one’s feelings appear to be detrimental for well-being given its positive association with NA, Clarity and Repair is a protective factor by virtue of its association with higher PA and lower NA. These results are consistent with the idea that higher scores on Clarity and Repair are positive for affective well-being (Palmer et al., 2002; Landa et al., 2006), as opposed to an excessive monitoring of our own feelings, which may lead to immersion in NA. This result is in line with the assumption that a moderate level of attention is adaptive, while higher levels can be counterproductive (Lischetzke and Eid, 2003; Fernández-Berrocal and Extremera, 2008; Lischetzke et al., 2012; Kong and Zhao, 2013).

Our third hypothesis was partially supported. Specifically, we only found Repair as a moderator in the relationship between teaching experience and PA. This means that teaching experience does not significantly decrease PA in teachers with higher levels of Repair. In contrast, low or moderate levels of Repair do not protect more highly experienced teachers from a reduction in their PA. These findings prompt the conclusion that teaching experience is prejudicial for affective well-being only for those who perceive themselves to be low or moderate in terms of Repair. These results are consistent with those of previous research showing that adequate levels of emotional regulation abilities are beneficial for personal and social success (Côté, 2014; Cabello and Fernández-Berrocal, 2015b; Gutiérrez-Cobo et al., 2017). Finally, no significant results were found for the Clarity or NA variables. The lack of a significant result for Clarity could be explained by the cascading model of EI (Joseph and Newman, 2010). This model suggests that each of the EI abilities fits into a progressive structure, in which Clarity is preceded by Attention, and Repair by Clarity. Therefore, high abilities to Repair emotions would also include the Clarity and Attention abilities (Fernández-Berrocal and Extremera, 2008; Côté, 2014; Cabello and Fernández-Berrocal, 2015b). Although we expected perceived EI to also moderate NA, previous studies have shown PA to be a more important aspect of well-being than NA (Kong and Zhao, 2013; Szczygieł and Mikolajczak, 2017). This is coherent with research findings suggesting that positive emotions have the power to undo the influence of negative emotions and that by increasing thought–action repertoires they can build consequential psychological and social resources (Fredrickson, 2013).

The present study has important practical implications. Given our results, different interventions should be implemented for teachers to promote their affective well-being and mitigate the negative effect caused by their experience. Currently, there is already evidence available regarding the positive effect of these type of interventions on adults on the general population and teachers, with EI training being considered as an effective intervention (Castillo et al., 2013; Bakker et al., 2016; Hodzic et al., 2017; van Wingerden et al., 2017). In addition, by using EI training to eliminate the aversive consequences of the years of teaching experience on PA, teachers could take advantages of the already known positive consequences of teaching experience on their effectiveness at work (Kini and Podolsky, 2016).

In spite of the relevance and novelty of our findings, there is an important limitation that is worth noting, that is, the lack of causal results. Future investigations should be aimed at looking for causal results by using longitudinal studies that analyze, for instance, how the age of the teacher influences the relationship between affective well-being and teaching experience, and the impact of intervention programs. Moreover, it would be interesting to examine how teachers’ implicit theories influence the relationship between EI and affective well-being (Cabello and Fernández-Berrocal, 2015a). In addition, although we were interested in analyzing the role of perceived EI, given the subjective nature of affective well-being, future research should include a measure of EI as an ability (Mayer et al., 2016). This would help to clarify the interaction between both constructs and, therefore, achieve a more solid understanding of the role of EI in affective well-being in the teaching profession and in other jobs with similar emotional and interpersonal demands.

## Conclusion

This study constitutes a step forward in understanding the protective variables of the affective well-being of teachers. Although years of teaching experience, which is an unchangeable variable, could be responsible for a decrease in the PA of teachers, this counterproductive effect could be mitigated by the teacher’s perception of high Mood Repair.

## Author Contributions

PF-B conceived of the study, participated in the data collection, analyzed the data, led preparation, and wrote the first draft of the manuscript. MG-C wrote the first draft of the manuscript. JR-C and RC conceived of the study, participated in the data collection, analyzed the data, and contributed to writing the manuscript. All authors contributed to the interpretation of data, helped to draft, and revised the manuscript and have read and approved the final manuscript.

## Conflict of Interest Statement

The authors declare that the research was conducted in the absence of any commercial or financial relationships that could be construed as a potential conflict of interest.
